# Checkpoint Kinase 1 (CHK1) Inhibition Enhances the Sensitivity of Triple-Negative Breast Cancer Cells to Proton Irradiation via Rad51 Downregulation

**DOI:** 10.3390/ijms21082691

**Published:** 2020-04-13

**Authors:** Changhoon Choi, Won Kyung Cho, Sohee Park, Sung-Won Shin, Won Park, Haeyoung Kim, Doo Ho Choi

**Affiliations:** 1 Department of Radiation Oncology, Samsung Medical Center, Seoul 06351, Korea; chchoi93@gmail.com (C.C.); wklove.cho@samsung.com (W.K.C.); psh3842@nate.com (S.P.); camuserik@gmail.com (S.-W.S.); wonro.park@samsung.com (W.P.); haeyoung0131.kim@samsung.com (H.K.); 2 Department of Radiation Oncology, Sungkyunkwan University School of Medicine, Seoul 06351, Korea

**Keywords:** proton therapy, triple-negative breast cancer, CHK1, Rad51

## Abstract

Due to a superior dose conformity to the target, proton beam therapy (PBT) continues to rise in popularity. Recently, considerable efforts have been directed toward discovering treatment options for use in combination with PBT. This study aimed to investigate the targeting of checkpoint kinase 1 (CHK1), a critical player regulating the G2/M checkpoint, as a promising strategy to potentiate PBT in human triple-negative breast cancer (TNBC) cells. Protons induced cell-cycle arrest at the G2/M checkpoint more readily in response to increased CHK1 activation than X-rays. A clonogenic survival assay revealed that CHK1 inhibition using PF-477736 or small interfering RNA (siRNA) enhanced the sensitivity toward protons to a greater extent than toward X-rays. Western blotting demonstrated that PF-477736 treatment in the background of proton irradiation increased the pro-apoptotic signaling, which was further supported by flow cytometry using annexin V. Immunofluorescence revealed that proton-induced DNA double-strand breaks (DSBs) were further enhanced by PF-477736, which was linked to the downregulation of Rad51, essential for the homologous recombination repair of DSBs. Direct inactivation of Rad51 resulted in enhanced proton sensitization. Collectively, these data suggest that targeting CHK1 may be a promising approach for improving PBT efficacy in the treatment of TNBC.

## 1. Introduction

Triple-negative breast cancer (TNBC) is a breast cancer subtype characterized by the lack of estrogen receptor (ER) and progesterone receptor (PR) expression, and absence of human epidermal growth factor receptor 2 (HER2) amplification. Moreover, TNBC constitutes approximately 10‒20% of all breast cancers [1,2]. Subsets of TNBC have demonstrated a remarkable response to chemotherapy, resulting in a better prognosis. In contrast, other subsets of TNBC have shown a poor response to treatment, consequently resulting in a poor prognosis [1]. Thus, several therapeutic agents such as poly ADP-ribose polymerase (PARP) inhibitors and PI3K/mTOR/AKT inhibitors are being tested as potential treatments for patients with TNBC [3,4,5]; however, chemotherapy remains the most beneficial form of treatment owing to the lack of approved targeted drugs.

Radiation therapy (RT) is a treatment method that locally destroys cancer cells using high energy photons or particles. Many preclinical and clinical studies have been performed to determine RT response in different molecular subtypes of breast cancer, and sensitivity to radiation seems to be higher in hormone-positive tumors as compared to that of HER2-type or TNBC [6]. To enhance the effects of RT in TNBC, several agents have been investigated in preclinical studies [5,7]. Cell cycle checkpoints are often deregulated in cancer cells, and thus, have been used as therapeutic targets. Mutations in TP53, a key protein regulating the G1 checkpoint, are more frequently seen in TNBCs than in ER-positive breast cancers. Thus, RT in combination with targeting of the G2/M checkpoint is an advantageous strategy for the treatment of TNBC. Furthermore, G2/M checkpoint abrogation using checkpoint kinase 1 (CHK1) inhibitors is known to increase the RT efficacy in preclinical models of pancreatic cancer [8], colorectal cancer [9], bladder cancer [10], and TNBC [11].

Proton beam therapy (PBT) is gaining popularity mainly due to the physical superiority of its Bragg peak, which leaves the healthy organs unaffected [12]. In breast cancer, this unique energy deposit profile can reduce heart and lung exposure to radiation, thereby potentially decreasing risk of cardiac toxicity and pneumonitis [12]. In radiobiology, the understanding of the advantages of proton beams over photon beams is still limited, although genetic alterations could modify the biological effectiveness of protons relative to that of photons in the lung, head and neck, liver, and breast cancer cells [13,14,15,16]. Proton beams induce more complex DNA damage, resulting in more prolonged DNA-damage repair and cell-cycle arrest than that observed in response to X-ray irradiation [17]. Furthermore, proton beams are capable of eliminating homologous recombination repair (HRR)-deficient tumor cells more effectively than photon beams [18]. Our recent study demonstrated that the sensitivity to protons varies across a panel of breast cancer cell lines, and that a cyclin D1/CDK4/RB1 axis may be implicated in determining proton sensitivity in TNBC cells. Considerable efforts are being directed towards discovering, or screening targeted drugs in combination with charged particles, including PBT [19]. This study aimed to evaluate the targeting of CHK1, a critical factor regulating the G2/M checkpoint, as a promising strategy to potentiate PBT in human TNBC cells.

## 2. Results

### 2.1. X-Rays and Protons Differently Affect ATR/CHK1 Activation in Human TNBC Cell Lines

**Figure 1:**
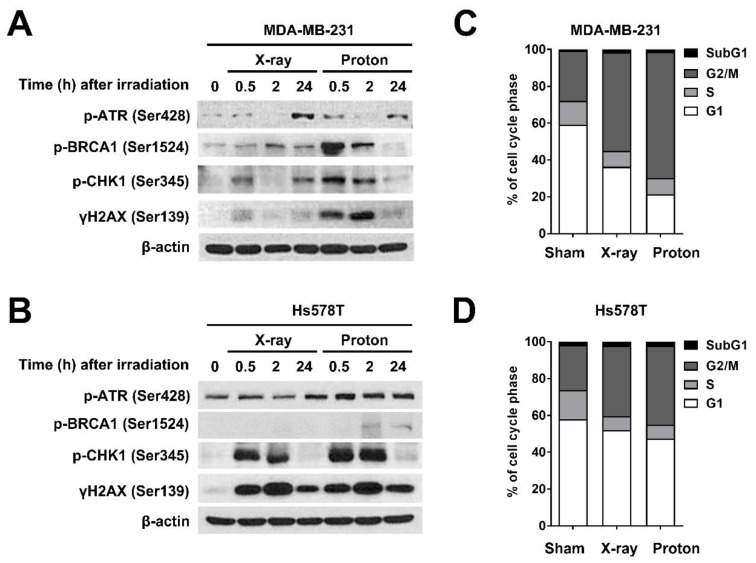
Differential checkpoint kinase 1 (CHK1) activation and cell cycle redistribution in response to irradiation with X-rays and protons in human triple-negative breast cancer (TNBC) cells. Western blotting showed differential activation of ataxia telangiectasia and rad3-related (ATR)/CHK1 signaling in response to X-ray and proton irradiation in MDA-MB-231 cells (**A**) and Hs578T cells (**B**). Cells were harvested at the indicated times after irradiation with 4 Gy of X-rays or protons. β-actin was used as a loading control. Cell-cycle distribution in MDA-MB-231 cells (**C**) and Hs578T (**D**) after irradiation with 4 Gy of X-rays or protons. Cells were harvested 24 h post-irradiation and subjected to flow cytometry.

To further understand the biological impact of proton irradiation in TNBC cells, we investigated the differential cell cycle checkpoint activation by X-rays and proton irradiation in two different TNBC cell lines, MDA-MB-231 and Hs578T. As both cell lines have a mutation in the TP53 gene, an essential gene for the G1/S checkpoint, we focused on the ataxia telangiectasia and rad3-related (ATR)/CHK1 signaling and the G2/M checkpoint. The samples were prepared 0.5 h, 2 h, and 24 h after exposure to 4 Gy of X-rays or protons. Western blotting revealed that in MDA-MB-231 cells, phosphorylation of ATR at Ser428 and BRCA1 at Ser1524 was strongly induced by protons at 0.5 h, with a concomitant increase in γH2AX expression, a surrogate marker for DNA double-strand breaks (DSBs) (Figure 1A). In addition, protons increased the phosphorylation of CHK1 at Ser345 to a greater extent than X-rays (Figure 1A). In Hs578T cells, there was a substantial increase in the phosphorylation of CHK1 and H2AX in response to both radiations. However, the difference between the two radiations was less pronounced in Hs578T cells than that in MDA-MB-231 cells (Figure 1B). Cell-cycle analysis showed that both radiations increased the cell population at the G2/M phase with a decrease in the G1 phase population in TNBC cells (Figure 1C,D). The effect of protons on the G2/M arrest was more prominent in MDA-MB-231 cells (Figure 1C) than that in Hs578T cells (Figure 1D). These data indicate that proton irradiation leads to the activation of CHK1 to a greater extent than that induced by X-rays, possibly due to more complex DNA damage, thereby resulting in a more pronounced G2/M arrest.

### 2.2. Pharmacological Inhibition of CHK1 Induces DNA Damage and Apoptosis in TNBC Cells

**Figure 2:**
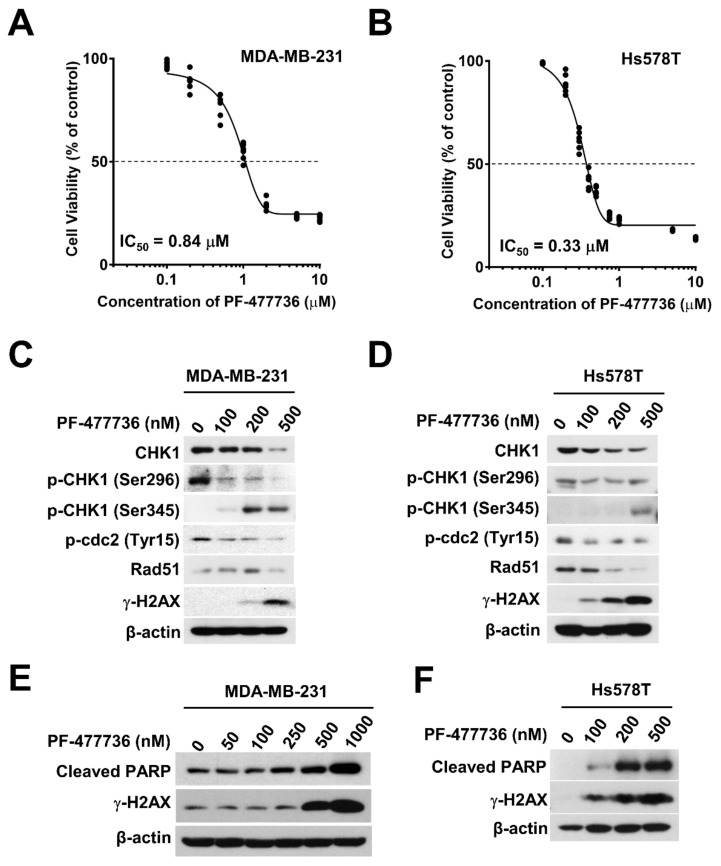
The cytotoxicity of the CHK1 inhibitor, PF-477736 is different in human TNBC cells. Cytotoxic effects of PF-477736 on MDA-MB-231 cells (**A**) and Hs578T cells (**B**). Cell proliferation was measured using the Cell Counting Kit-8 (CCK-8) assay kit after 72 h of PF-477736 treatment. Concentration-dependent inhibition of CHK1 activity in response to PF-477736 treatment in MDA-MB-231 cells (**C**) and Hs578T cells (**D**). Cells were incubated with the indicated concentrations of PF-477736 for 48 h. β-actin was used as a loading control. Concentration-dependent cellular damage in response to PF-477736 treatment in MDA-MB-231 cells (**E**) and Hs578T cells (**F**). Cleaved polymerase (PARP) and γ-H2AX were used as markers of apoptosis and DNA damage. β-actin was used as a loading control.

Based on our findings that protons activated CHK1 and the G2/M checkpoint more readily than X-rays (Figure 1A), we targeted CHK1 using a pharmacological inhibitor and evaluated its effect on proton sensitization. Before investigating the efficacy of proton irradiation with respect to CHK1 inhibition in human breast cancer cells, we tested the cytotoxicity of a selective pharmacological CHK1 inhibitor, PF-477736 in TNBC cells. The results of the Cell Counting Kit-8 (CCK-8) assay showed a dose-dependent inhibition of cell viability in response to PF-477736 treatment, with the half maximal-inhibitory concentration (IC_50_) being 0.84 μM and 0.33 μM for MDA-MB-231 and Hs578T cells, respectively (Figure 2A,B). The inhibitory effect of PF-477736 on CHK1 activity was evident from the decreased levels of phospho-Ser296 CHK1 and an increase in phospho-Ser345 CHK1 levels in MDA-MB-231 cells (Figure 2C). Inhibition of CHK1 activity impairs CHK1 autophosphorylation at Ser296, stabilizes cdc25a, and potentiates ATR-mediated CHK1 phosphorylation at Ser345, which provides a docking site for E3 ligase, leading to ubiquitin-proteasome-dependent degradation of CHK1 (Figure 2C,D). Additionally, PF-477736 also decreased the expression of phospho-Tyr15-cdc2 (a target of CHK1) and Rad51. These data are consistent with previous results on another CHK1 inhibitor, AZD7762 [8]. PF-477736 increased DNA damage and apoptosis in a dose-dependent manner, which was assessed based on the increase in the levels of γH2AX and cleaved PARP (Figure 2E,F). Moreover, Hs578T cells demonstrated a DNA damage response and apoptotic cell death at 100 nM of PF-477736, whereas MDA-MB-231 cells did not. Another TNBC cell line, MDA-MB-453, also showed sensitivity to PF-477736 in a manner similar to Hs578T (Figure S1A,B), suggesting that MDA-MB-231 cells are relatively resistant to the CHK1 inhibitor.

### 2.3. CHK1 Inhibition in Response to PF-477736 Treatment Sensitizes TNBC Cells to Proton Irradiation

**Figure 3:**
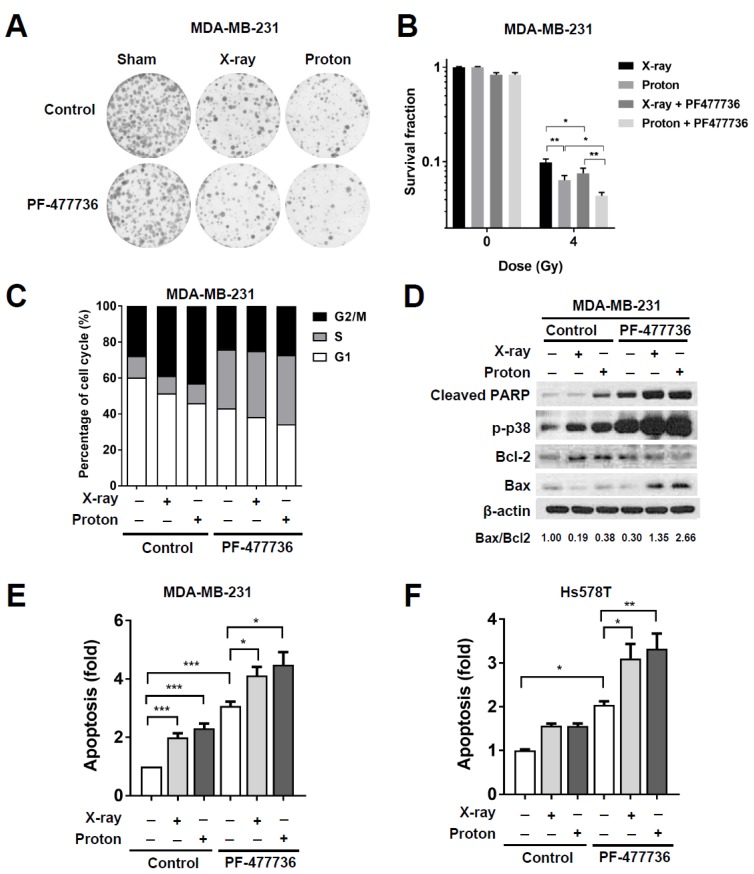
CHK1 inhibition in response to PF-477736 treatment sensitizes TNBC cells to proton irradiation. (**A**) Effect of co-treatment with PF-477736 and 4 Gy of X-rays or protons on clonogenic survival. MDA-MB-231 cells were pre-treated with 100 nM PF-477736 for 3 h, followed by irradiation with 4 Gy of X-rays or protons. Colonies were stained with crystal violet after 14 days; (**B**) Quantification of survived colonies. Data are shown as mean ± S.D. from two independent experiments. * *p* < 0.05; ** *p* < 0.01; (**C**) Cell-cycle distribution after combinatorial treatment with PF-477736 and X-rays or protons. MDA-MB-231 cells pre-treated with 500 nM PF-477746 for 3 h were irradiated with 4 Gy of X-rays or protons and were harvested 24 h after irradiation for flow cytometric analysis. X-ray and proton irradiations led to a significant increase in the cell population at the G2/M phase, and their combination with PF-477736 increased the cell population at the S phase; (**D**) Western blotting showed that pre-treatment of 500 nM PF-477736 for 3 h, followed by 4 Gy radiation increased apoptotic signaling, as compared to that seen upon irradiation alone. Cells were harvested 72 h post-irradiation. β-actin was used as a loading control. Densitometric analysis showed increased Bcl-2-associated X (Bax)/B-cell lymphoma 2 (Bcl-2) ratio after the combined treatment. Enhanced apoptosis in response to combinatorial treatment with 500 nM PF-477736 and 4 Gy radiation in MDA-MB-231 cells (**E**) and Hs578T cells (**F**). MDA-MB-231 cells and Hs578T cells were pre-treated for 3 h with 500 nM and 100 nM of PF477736, respectively, followed by irradiation with 4 Gy of X-rays or protons. Cells were harvested 72 h post-irradiation and apoptotic population was determined as described in Materials and Methods. Quantification data were shown. Data are shown as mean ± S.D. from three independent experiments * *p* < 0.05; ** *p* < 0.01; *** *p* < 0.001.

In order to determine the effect of CHK1 inhibition on the sensitivity to radiation, we performed a clonogenic survival assay. MDA-MB-231 cells were pre-treated with 100 nM PF-477736 for 3 h and then exposed to 4 Gy of X-rays or protons. The surviving colonies were stained and counted after 14 d of irradiation. Both X-rays and protons effectively inhibited the clonogenic survival of MDA-MB-231 cells; protons significantly affected the survival as compared to X-rays (*p* < 0.01; Figure 3A,B). The effect of the combination of PF-477736 and irradiation was significant in proton-irradiated cells (*p* < 0.01) than that in X-ray-irradiated cells (*p* < 0.05; Figure 3B). Cell counts revealed significantly lower clonogenic survival of MDA-MB-231 cells in response to combinatorial treatment with 100 nM PF-477736 and proton irradiation than with X-ray irradiation (*p* < 0.01; Figure 3B). Next, the effect of PF-477736 on radiation-induced cell-cycle redistribution was determined (Figure 3C). As shown in Figure 1C, proton irradiation led to a marked arrest at the G2/M phase. PF-477736 dramatically increased the cell population in the S-phase, indicating the abrogation of radiation-induced G2/M arrest (Figure 3C).

Furthermore, we found that treatment with 500 nM PF-477736 increased the levels of pro-apoptotic proteins such as Bcl-2-associated X (Bax), phospho-p38, and cleaved PARP in MDA-MB-231 cells, which were further enhanced in response to combinatorial treatment with PF-477736 and 6 Gy of either X-rays or protons (Figure 3D). The densitometric analysis showed that the Bax/B-cell lymphoma 2 (Bcl-2) ratio was the highest upon co-treatment with protons and PF-477736. Analysis of apoptosis using annexin V/propidium iodide double-staining confirmed that apoptosis of MDA-MB-231 cells was significantly increased in response to irradiation with either X-rays (*p* < 0.001) or protons (*p* < 0.001; Figure 3E). Further, treatment with 500 nM PF-477736 increased the number of apoptotic cells (*p* < 0.001), which was further increased upon combination with the two radiations (*p* < 0.05). However, no significant difference between the combinatorial treatments using either proton or X-ray irradiation was observed with respect to the number of apoptotic cells (Figure 3E). In Hs578T cells, treatment with 100 nM PF-477736 induced apoptosis (*p* < 0.05) and both radiations enhanced apoptosis when combined with 100 nM PF-477736 (*p* < 0.05 and *p* < 0.01 for X-rays and protons, respectively; Figure 3F). MDA-MB-453 cells also showed enhanced apoptotic cell death in response to combinatorial treatment with 100 nM PF-477736 and protons (Figure S1C).

### 2.4. CHK1 Knockdown via Small Interfering RNA (siRNA) Treatment Sensitizes MDA-MB-231 Cells to Proton Irradiation

**Figure 4:**
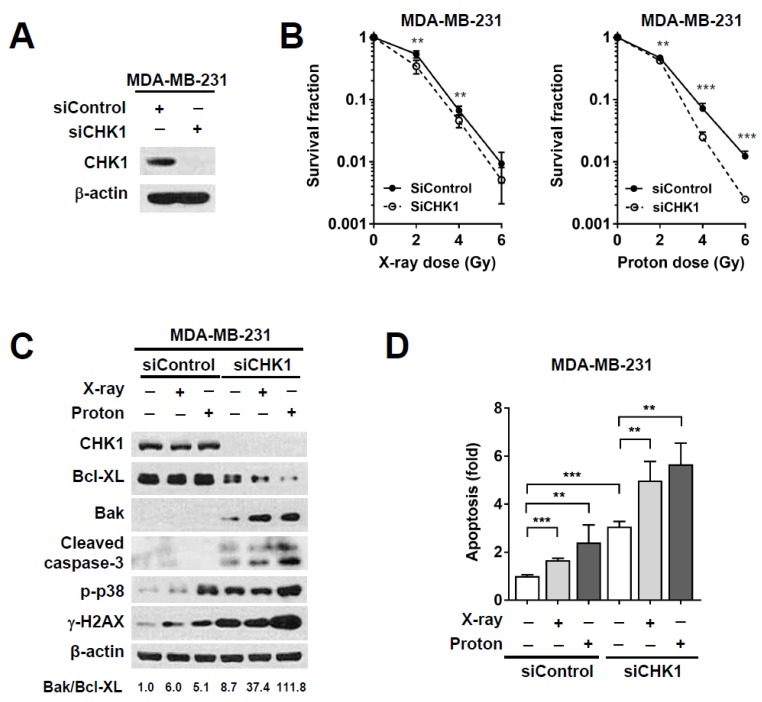
CHK1 silencing using small interfering RNA (siRNA) sensitizes MDA-MB-231 cells to proton irradiation. (**A**) siRNA-mediated CHK1 knockdown was confirmed using western blotting. MDA-MB-231 cells were treated with 10 nM siRNA, and CHK1 levels were determined after 24 h. β-actin was used as loading control; (**B**) The clonogenic survival assay showed that siRNA-mediated CHK1 knockdown resulted in enhanced proton radiosensitivity of MDA-MB-231 cells. Data are shown as mean ± S.D. from two independent experiments. ** *p* < 0.01; *** *p* < 0.001; (**C**) Western blot showed that CHK1 knockdown using siRNA further increased the radiation-induced pro-apoptotic signaling. Cells were treated with 10 nM siRNA for 24 h, followed by irradiation with 4 Gy of X-rays or protons. Cells were harvested 72 h post-irradiation. β-actin was used as a loading control. Densitometric analysis showed increased Bak/Bcl-XL ratio after co-treatment with CHK1 siRNA and protons; (**D**) Flow cytometric analysis revealed that apoptosis was enhanced in response to combinatorial treatment with CHK1 siRNA and 4 Gy of proton irradiation. Data are shown as mean ± S.D. from two independent experiments. ** *p* < 0.01; *** *p* < 0.001.

Increased S-phase cells upon treatment with PF-477736 alone was not different from those upon its combination treatment with radiations (Figure 3C), suggesting that PF-477736 might have non-specific toxicity. To determine whether proton radiosensitization by PF-477736 may be off-target effects, we performed gene silencing experiments for CHK1. Western blotting confirmed siRNA-mediated knockdown of CHK1 in MDA-MB-231 cells (Figure 4A). Radiosensitization in response to CHK1 knockdown was determined using the clonogenic assay (Figure 4B). As compared to the control siRNA, CHK1 siRNA lowered the fraction of survived cells in irradiated MDA-MB-231 cells, which was further evident upon proton irradiation (Figure 4B). With respect to pharmacological inhibition, siRNA-mediated CHK1 knockdown enhanced apoptosis, as evidenced by the increased levels of cleaved caspase-3. As compared to the control siRNA, CHK1 siRNA increased the expression of the pro-apoptotic protein Bak with a concomitant decrease in the expression of the anti-apoptotic protein Bcl-XL. In addition, the ratio of Bak/Bcl-XL was the highest in the cells co-treated with siCHK1 and protons (Figure 4C). The effects of CHK1 knockdown on apoptotic cell death were similar to those of PF-477736; CHK1 knockdown enhanced proton-induced apoptosis, as evidenced by annexin V staining in flow cytometry (Figure 4D). Together, these data suggest that CHK1 inhibition by either a small molecule inhibitor or siRNA increased proton radiosensitivity of TNBC cells via induction of apoptosis.

**Figure 5:**
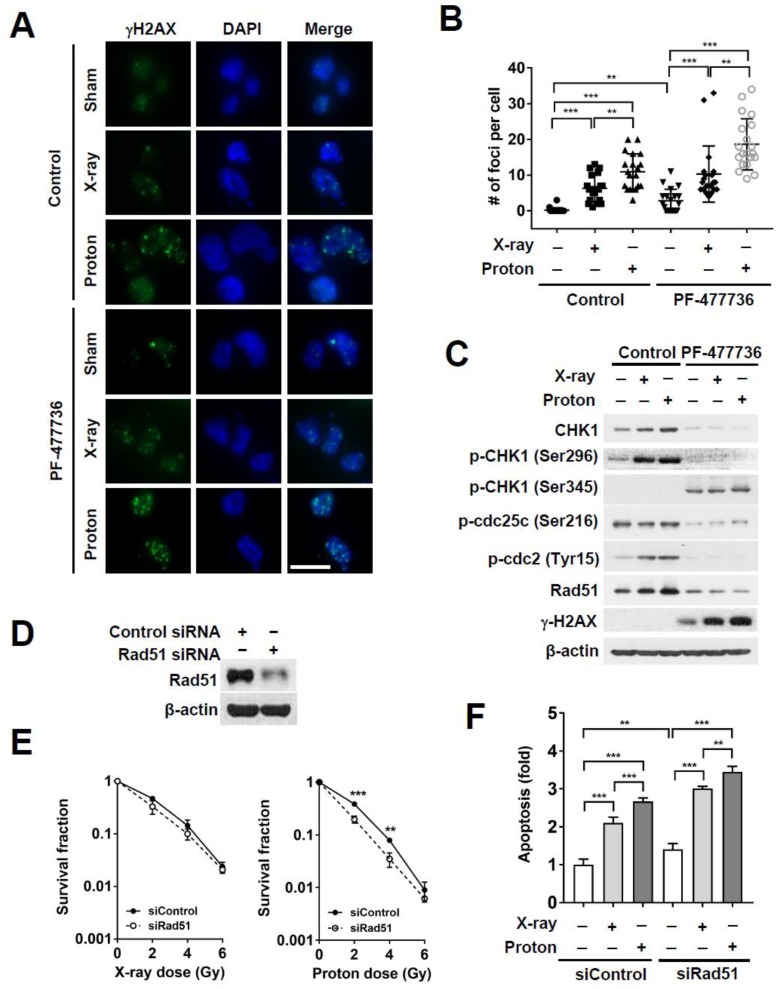
CHK1 inhibition in response to PF-477736 treatment augments proton irradiation-induced DNA damage in MDA-MB-231 cells via Rad51 downregulation. (**A**) Representative immunofluorescence images showing enhanced proton-induced DNA damage upon CHK1 inhibition. MDA-MB-231 cells were pre-treated with 500 nM PF-477736 for 3 h, followed by irradiation with 4 Gy of X-rays or protons. After 24 h of irradiation, cells were fixed and probed using the γ-H2AX antibody (green) and stained with 4′, 6-diamidino-2-phenylindole (DAPI) (blue). The scale bar indicates 10 μm; (**B**) Quantification data showing a significant increase in the number of γ-H2AX foci in the nucleus upon pre-treatment with 500 nM PF-477736 for 3 h, followed by irradiation with 4 Gy of protons. Data are shown as mean ± S.D (n = 55) from two independent experiments. ** *p* < 0.01; *** *p* < 0.001; (**C**) Induction of Rad51 expression in response to proton irradiation was suppressed upon pre-treatment with PF-477736; (**D**) Western blotting verified siRNA-mediated knockdown of Rad51 in MDA-MB-231 cells. β-actin was used as a loading control; (**E**) Knockdown of Rad51 via siRNA increased the radiosensitization of MDA-MB-231 cells to protons as compared to that observed upon using control siRNA; however, it did not affect the radiosensitization to X-rays. Data are shown as mean ± S.D. from two independent experiments. ** *p* < 0.01; *** *p* < 0.001; (**F**) Knockdown of Rad51 via siRNA enhanced proton-induced apoptosis as compared to that observed upon using control siRNA. Data are shown as mean ± S.D. from two independent experiments. ** *p* < 0.01; *** *p* < 0.001.

CHK1 plays a critical role in the DNA damage response as well as checkpoint activation. In order to understand the effect of CHK1 inhibition on proton-induced DNA damage, we evaluated the number of γH2AX foci in irradiated MDA-MB-231 cells in the presence or absence of PF-477736. Cells were fixed and stained with anti-γ-H2AX antibodies 24 h post-irradiation. Immunofluorescence showed that independent treatment with PF-477736 increased the number of γ-H2AX foci, as compared to those seen in the untreated control cells (*p* < 0.01; Figure 5A,B). Both radiations dramatically increased γH2AX foci formation (*p* < 0.001); proton irradiation induced γ-H2AX foci formation to a greater extent than X-ray irradiation (*p* < 0.01). Combinatorial treatment using PF-477736 and radiation further augmented the number of γ-H2AX foci, as compared to either treatment alone (*p* < 0.001); a greater number of γ-H2AX foci were seen in proton-irradiated cells than in X-ray-irradiated cells (*p* < 0.01; Figure 5A,B). Immunofluorescence of γ-H2AX at 30 min post-irradiation showed that both radiations increased γH2AX foci formation, but PF-477736 did not augment the radiation-induced γH2AX foci formation at such an early time point (Figure S2).

Both X-ray and proton irradiation increased the levels of CHK1 and phospho-cdc2 (Tyr15). However, PF-477736 treatment abolished the increase in levels of these proteins (Figure 5C). The expression of Rad51, a DNA damage repair protein, was markedly reduced in PF-477736-treated cells, regardless of the radiation treatment (Figure 5C). In order to determine whether Rad51 might be implicated in proton sensitization upon CHK1 inhibition, we knocked down Rad51 using RNA interference (Figure 5D). The clonogenic survival assay showed that Rad51 siRNA enhanced radiosensitization of MDA-MB-231 cells to proton irradiation to a greater extent than X-ray irradiation, relative to that observed upon treatment with control siRNA (Figure 5E). Apoptosis assay confirmed that Rad51 knockdown further augmented radiation-induced apoptosis (*p* < 0.001); in the background of Rad51 knockdown, proton irradiation resulted in a higher number of apoptotic populations than X-ray irradiation (*p* < 0.01; Figure 5F). Furthermore, treatment with B02, a selective Rad51 inhibitor, increased the level of DNA damage and apoptosis (Figure S3A), leading to proton sensitization, as evidenced by the results of the clonogenic survival assay (Figure S3B,C). These data suggest that the downregulation of Rad51 may be associated with a causal link between CHK1 inhibition and proton sensitization.

## 3. Discussion

CHK1 is a serine/threonine-protein kinase that plays an important role in maintaining genomic stability. It is phosphorylated in response to DNA damage and facilitates two key functions, cell cycle arrest and DNA repair through the homologous recombinational repair (HRR) pathway [4,20,21]. CHK1 is frequently deregulated in various types of cancers, and its inhibition in combination with antimetabolites or DNA-damaging agents enhances cytotoxicity [22]. CHK1 inhibitors potentiate RT in various preclinical models [8,9,10,23], especially in being effective radiosensitizers in p53-deficient tumor cells by targeting the G2/M checkpoint [8,22]. In head and neck squamous cell carcinoma (HNSCC) cells, PF-477736 radiosensitized human papillomavirus (HPV)-positive cancer cells lacking TP53 by abrogating radiation-induced G2/M arrest. However, it did not show any such effect in normal fibroblasts [24]. Furthermore, CHK1 inhibitors have been shown to sensitize p53-expressing colorectal cancer HCT116 cells to radiation [9]. Although enhanced efficacy of conventional photon RT by CHK1 inhibitors has been well described, thus far, no data are available regarding the effect of proton beams. In this study, we directly compared the effectiveness protons and X-rays with respect to CHK1 inhibition in various TNBC cells.

TNBC is a heterogeneous group of tumors, which have the worst prognosis among breast cancer subtypes [1]. There are several hypotheses regarding the resistance of TNBC to treatments, including radiotherapy. Moreover, TP53 mutation is more frequently observed in TNBC than in other breast cancer subtypes and is associated with radioresistance. For example, TP53 mutation is seen in 30% of all breast cancers, and is most frequently observed in the basal-like type of TNBC (88%), whereas it is less common in the luminal A subtype (17%) [25]. TP53 mutations are associated with radioresistance; this is mediated via activation of DNA repair genes including Rad51, or by inhibition of p53-induced apoptosis [26]. Further, TP53 mutations in TNBC also result in the loss of the G1 checkpoint, as well as the dependence on CHK1 for responding to DNA damage. Moreover, CHK1 is significantly overexpressed in TNBC tissues as compared to that in non-TNBC tissues [27]. Thus, targeting CHK1 in TNBC might be a plausible approach to enhance the efficacy of RT.

Proton therapy biologically shows a 10% higher effectiveness than conventional photon therapy, and the current use of proton therapy largely relies on its physical superiority, such as the Bragg peak [28]. Accumulating evidence suggests that there is a difference in the biological efficacy between proton and photon therapies [13,15,16,18,29,30]. In the form of a particle beam, protons cause more DNA damage than photons, as photons lack mass. Recent preclinical trial data suggest that HRR appears to be crucial for proton-induced DNA damage repair, whereas, non-homologous end joining (NHEJ) is important for photon-induced DNA damage repair [18]. Impairment of HRR is one of the mechanisms by which CHK1 inhibitors function in cancer cells. Thus, we hypothesized that CHK1 inhibition might be more effective in combination with proton therapy than with photon therapy. The clonogenic survival assay showed that the surviving colony numbers of MDA-MB-231 cells were significantly reduced in response to combination treatment (PF-477736 and radiation) as compared to PF-477736 treatment or radiation alone. As expected, the combination of PF-477736 treatment along with proton therapy showed a pronounced effect on clonogenic survival than X-rays. Considering that PF-477736 has a strong affinity for CHK2, in a manner similar to other CHK1 inhibitors [22], we knocked down CHK1 using siRNA and confirmed that the proton-radiosensitizing effect of PF-477736 is mainly due to the selective inhibition of CHK1 activity.

Morgan et al. [8] reported that CHK1 inhibition using AZD7762 not only abrogates the G2 checkpoint but also impairs HRR, as indicated by the reduced formation of Rad51 foci and persistent γH2AX expression. Consistently, our data showed that PF-477736 abrogated the G2/M checkpoint and accumulated DNA damage, as evidenced by the reduced Rad51 expression and increased γH2AX levels. Immunofluorescence revealed that combinatorial treatment with PF-477736 and proton therapy generated more γH2AX foci than combinatorial treatment with PF-477736 and X-rays, suggesting that PF-477736 might be involved in enhancing proton-induced DNA DSBs. CHK1 phosphorylates DNA repair proteins, including Rad51, FANCD2, and FANCE, and activates HRR [22]. Amongst these, Rad51 is an essential protein for HRR, and its recruitment to the sites of DNA damage triggers the initiation of HRR. Knockdown of Rad51 or Rad51 inhibitor treatment enhanced proton-mediated clonogenic cell death and apoptosis of TNBC cells, supporting the hypothesis that the downregulation of Rad51 in response to PF-477736 treatment might result in enhanced sensitivity to proton therapy. The radiosensitizing effect of PF-477736 with respect to X-rays was relatively weak, which may be attributed to residual Rad51 expression.

Our data support previous findings that HRR-deficient tumor cells are more susceptible to proton irradiation than to photon irradiation [18]. TNBC is frequently associated with mutations in DNA damage repair-associated genes, which are being utilized as therapeutic targets [31]. For example, BRCA mutations have been reported in 20% of the TNBC cases, and the BRCA1 promoter CpG island methylation is highly prevalent in patients with TNBC [32]. Synthetic lethality in response to PARP inhibitors, such as olaparib, has been tested in BRCA-deficient breast cancer patients [33]. Our findings suggest that proton therapy could be considered a superior alternative to conventional X-ray therapy for the elimination of residual TNBC cells, especially those with an impaired HRR pathway. CHK1 is one of the therapeutic target candidates in TNBC, which has no approved targeted therapy. Our data suggest that CHK1 inhibition sensitizes TNBC cells to proton therapy both by abrogating the radiation-induced G2/M arrest and by impairing HRR through Rad51 downregulation. To our knowledge, this study is the first of its kind to provide evidence for increased efficacy of proton therapy when used in combination with a CHK1 inhibitor in TNBC cells. Further investigation should be made regarding the use of the combinatorial treatment using proton therapy and CHK1 inhibition as an alternative strategy for the treatment of refractory TNBC.

## 4. Materials and Methods

### 4.1. Cell Culture

Three human TNBC cell lines, MDA-MB-231, Hs578T, and MDA-MB-453, were used in this study. MDA-MB-231 cells were purchased from the American Type Culture Collection (ATCC, Manassas, VA, USA), and Hs578T cells and MDA-MB-453 cells were purchased from the Korean Cell Line Bank (Seoul National University, Seoul, Korea). MDA-MB-231 and MDA-MB-453 cells were cultured in Dulbecco′s Modified Eagle’s Medium (DMEM) supplemented with 10% fetal bovine serum (FBS), and Hs578T cells were cultured in DMEM supplemented with 10% FBS and 25 mM 4-(2-hydroxyethyl)-1-piperazineethanesulfonic acid (HEPES). Cultures were maintained in a humidified atmosphere comprising 95% air and 5% CO2 at 37 °C.

### 4.2. Reagents and Antibodies

Anti-phospho-H2AX (Ser139) antibodies were purchased from Millipore (Burlington, MA, USA). Anti-cleaved PARP, anti-cleaved caspase-3, anti-phospho-p38 MAPK (Thr180/Tyr182), anti-CHK1, anti-phospho-CHK1 (Ser345 and Ser296), anti-phospho-cdc2 (Tyr15), anti-phospho-BRCA1 (Ser1524), anti-Bcl2, anti-Bax, anti-Bak, anti-Bcl-XL, and anti-Rad51 antibodies were purchased from Cell Signaling Technology (Danvers, MA, USA). Anti-β-actin antibodies were purchased from Sigma Aldrich (St. Louis, MO, USA). Anti-Rad51 antibodies were purchased from Santa Cruz Biotechnology (Santa Cruz, CA, USA). For gene knockdown experiments, control siRNA (sc-37007), CHK1 siRNA (sc-29269), and Rad51 siRNA (sc-36361) were purchased from Santa Cruz Biotechnology. RNAiMax was purchased from Invitrogen (Carlsbad, CA, USA). PF-477736, a selective CHK1 inhibitor, and B02, a Rad51 inhibitor, were purchased from Sigma Aldrich.

### 4.3. Cell Viability Assay

The Cell Counting Kit-8 (CCK-8, Dojindo Laboratories, Kumamoto, Japan) was used to determine cell viability according to the manufacturer’s instructions. Briefly, MDA-MB-231 cells and Hs578T cells were seeded at a density of 1 × 10^3^ cells/well into a 96-well plate, and on the next day were treated with various concentrations of PF-477736 dissolved in dimethyl sulfoxide (DMSO). Cells were incubated with the CCK-8 solution 72 h post-treatment with PF-477736, and cell viability was determined by measuring the absorbance at 450 nm using the SpectraMax i3 microplate reader (Molecular Devices, Sunnyvale, CA, USA).

### 4.4. Irradiation Experiments

X-ray beams and proton beams were made incident using a linear accelerator Varian Clinac 6EX (Varian Medical Systems, Palo Alto, CA, USA), and a proton therapy system (Sumitomo Heavy Industries, Tokyo, Japan) respectively, at the Samsung Medical Center in Seoul, Korea [34]. For proton irradiation, cell plates were positioned in the center of the spread-out Bragg peak (SOBP) measuring 11.2 cm within a field collimated by 18 × 12 cm Brass blocks. Cells were irradiated with graded doses of 230 MeV protons, for which the beam range was 22.8 cm (distal 90%) [35]. The absolute proton beam dose was verified to an accuracy of 1% for proton therapy, in accordance with TRS-398. For X-ray irradiation, cells were placed under a 2 cm thick solid water phantom at a source surface distance of 100 cm and a field size of 30 × 30 cm. The absolute X-ray dose was verified in accordance with TG-51 using the Gafchromic film to an accuracy of 1%.

### 4.5. Clonogenic Survival Assay

Cells were seeded at a low density (800–3200 cells/60 mm dish) 24 h before irradiation and were irradiated with various doses of X-rays or protons. After incubation for 14 d, surviving colonies consisting of more than 50 cells were visualized by staining with 0.5% crystal violet (Sigma-Aldrich) and were then counted. The survival fraction (SF) was calculated as the ratio of the plating efficiency (ratio of the number of surviving colonies to the number of seeded cells) of the irradiated cells to the plating efficiency of un-irradiated cells in order to quantify the cell survival [36].

### 4.6. Immunoblotting

Cells were lysed using modified RIPA buffer (20 mM Tris (pH 8.0), 137 mM NaCl, 10% glycerol, 1% Nonidet *p*-40, 10 mM EDTA, 100 mM NaF, 1 mM phenylmethylsulfonyl fluoride, and 10 mg/mL leupeptin), and the supernatant was collected after centrifugation at 15,871 *g* for 15 min. Protein concentration was measured using the Bio-Rad protein assay reagent (Bio-Rad, Richmond, CA, USA), according to the manufacturer′s instructions. Equal amounts of protein were subjected to sodium dodecyl sulfate-polyacrylamide gel electrophoresis (SDS-PAGE). Separated proteins were transferred onto nitrocellulose membranes (Bio-Rad); blots were blocked overnight with 5% skimmed milk prepared in PBS, pH 7.4 at 4 °C and probed with primary antibodies overnight. Protein bands were detected using the Amersham enhanced chemiluminescence detection reagents (GE Healthcare, Piscataway, NJ, USA).

### 4.7. Flow Cytometry

Flow cytometry was performed in order to measure the number of apoptotic cells. Harvested cells were stained with annexin V-fluorescein isothiocyanate (FITC) (BD Pharmingen, San Diego, CA, USA) and 2 μg/mL PI in the annexin V binding buffer (10 mM HEPES, pH 7.4, 140 mM NaCl, 2.5 mM CaCl_2_) for 15 min at 37 °C in the dark. The number of apoptotic cells was quantified using BD FACSVerse and the BD FACSuite software.

### 4.8. Immunofluorescence

Image acquisition and analyses were conducted as described previously [37]. Briefly, 2 × 104 MDA-MB-231 cells were seeded on a cover slip (Marinfild Inc., Rochester, NY, USA) a day before irradiation. Cells were pre-treated with 500 nM PF-477736 for 3 h, exposed to photon or proton beams, and then fixed with 4% formaldehyde at 24 h. After permeabilization using 0.01% Triton X-100, and blocking with 2% bovine serum albumin (BSA) for 30 min, cells were incubated with the phospho-S193 H2AX antibody for 2 h, followed by incubation with the Alexa Fluor 488-conjugated secondary antibody (Life Technologies, Paisley, UK) and 4′,6-diamidino-2-phenylindole (DAPI) for 30 min at room temperature. After mounting the cells using the SlowFade anti-fade reagent (Molecular Probes, Eugene, OR, USA), images were acquired using a fluorescence microscope (Zeiss Observer D1, Carl Zeiss Co., Ltd., Jena, Germany).

### 4.9. Gene Knockdown Experiments

Small interfering RNAs (siRNAs) were used to knockdown CHK1 and Rad51 proteins. For transfection, 1 × 105 MDA-MB-231 cells were seeded in 6-well plates and incubated with a mixture of 10 nM siRNA and Lipofectamine RNAiMax (1:1 ratio) for 4 h, and then the culture medium was replaced with fresh medium. Reduction in the expression of the proteins of interest was confirmed using immunoblotting.

### 4.10. Statistical Analysis

Data are presented as the mean ± standard deviation (S.D.). Statistical analysis was performed using GraphPad Prism 7.02. Significance of differences between two groups was determined using the unpaired, two-tailed Student’s t-test. For a comparison of more than two groups, one-way ANOVA with Tukey’s multiple comparison test was used. *p* < 0.05 was considered to be statistically significant.

## Supplementary Materials

The following are available online at https://www.mdpi.com/1422-0067/21/8/2691/s1.

## Abbreviations

ANOVA	Analysis of variance
ATR	Ataxia telangiectasia and rad3-related
Bax	Bcl-2-associated X
Bcl-2	B-cell lymphoma 2
BSA	Bovine serum albumin
CHK1	Checkpoint kinase 1
DAPI	4′,6-diamino-2-phenylindole
FITC	Fluorescein isothiocyanate
PBT	Proton beam therapy
DSB	Double strand break
HRR	Homologous recombination repair
NHEJ	Non-homologous end joining
PARP	Poly(ADP-ribose)polymerase
RT	Radiation therapy
siRNA	Small interfering ribonucleic acid
TNBC	Triple negative breast cancer
