# Supplementary material for: Checkpoint Kinase 1 (CHK1) Inhibition Enhances the Sensitivity of Triple-Negative Breast Cancer Cells to Proton Irradiation via Rad51 Downregulation
Source: Int J Mol Sci. 2020 Apr 13;21(8):2691. doi: 10.3390/ijms21082691 (PMC7215565; doi:10.3390/ijms21082691)
Supplement: Supplementary file 1 [file ijms-21-02691-s001.pdf]

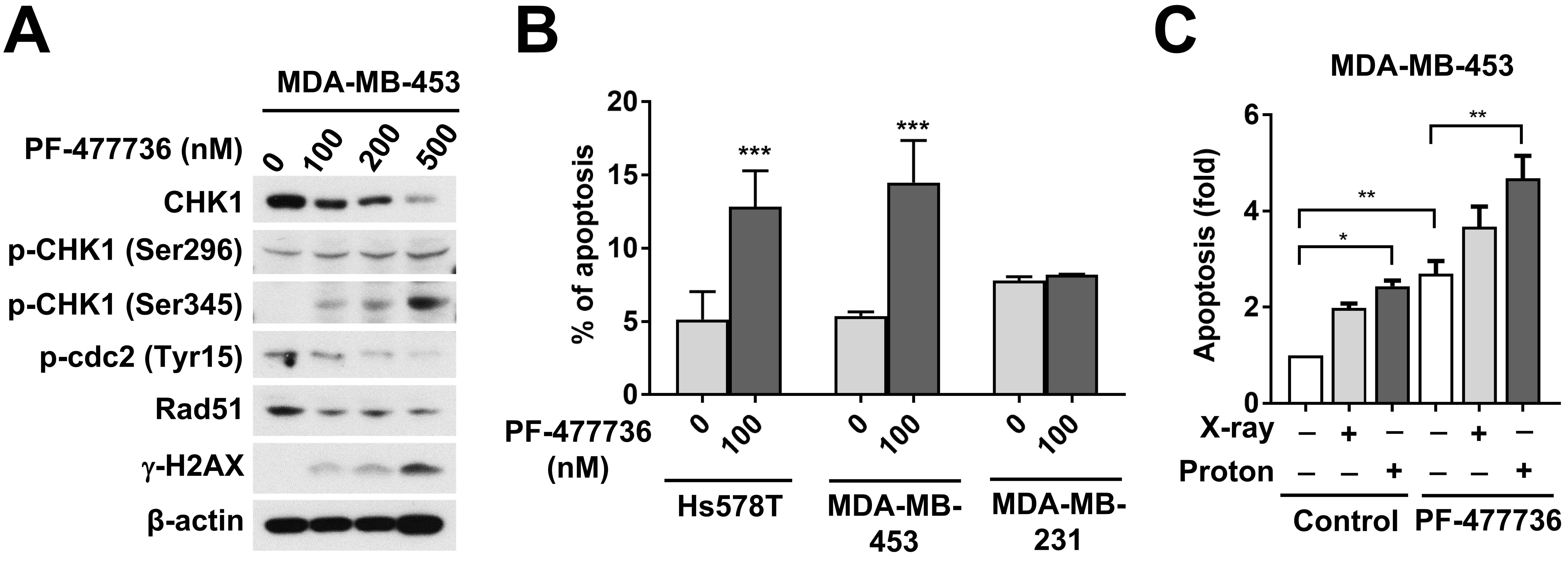

Figure S1. Effects of PF-477736 on a human TNBC cell line, MDA-MB-453 cells. (**A**) PF-477736 inhibited CHK1 activity in a concentration-dependent manner. (**B**) Comparison of PF-477736-induced apoptosis in three TNBC cell line, MDA-MB-231 cells were more resistant to PF-477736 than Hs578T cells and MDA-MB-453 cells in terms of apoptosis. ***$p<0.001$. (**C**) Pretreatment with 100 nM PF-477736 further enhanced proton-induced apoptosis in MDA-MB-453 cells. *$p<0.05$; **$p<0.01$.

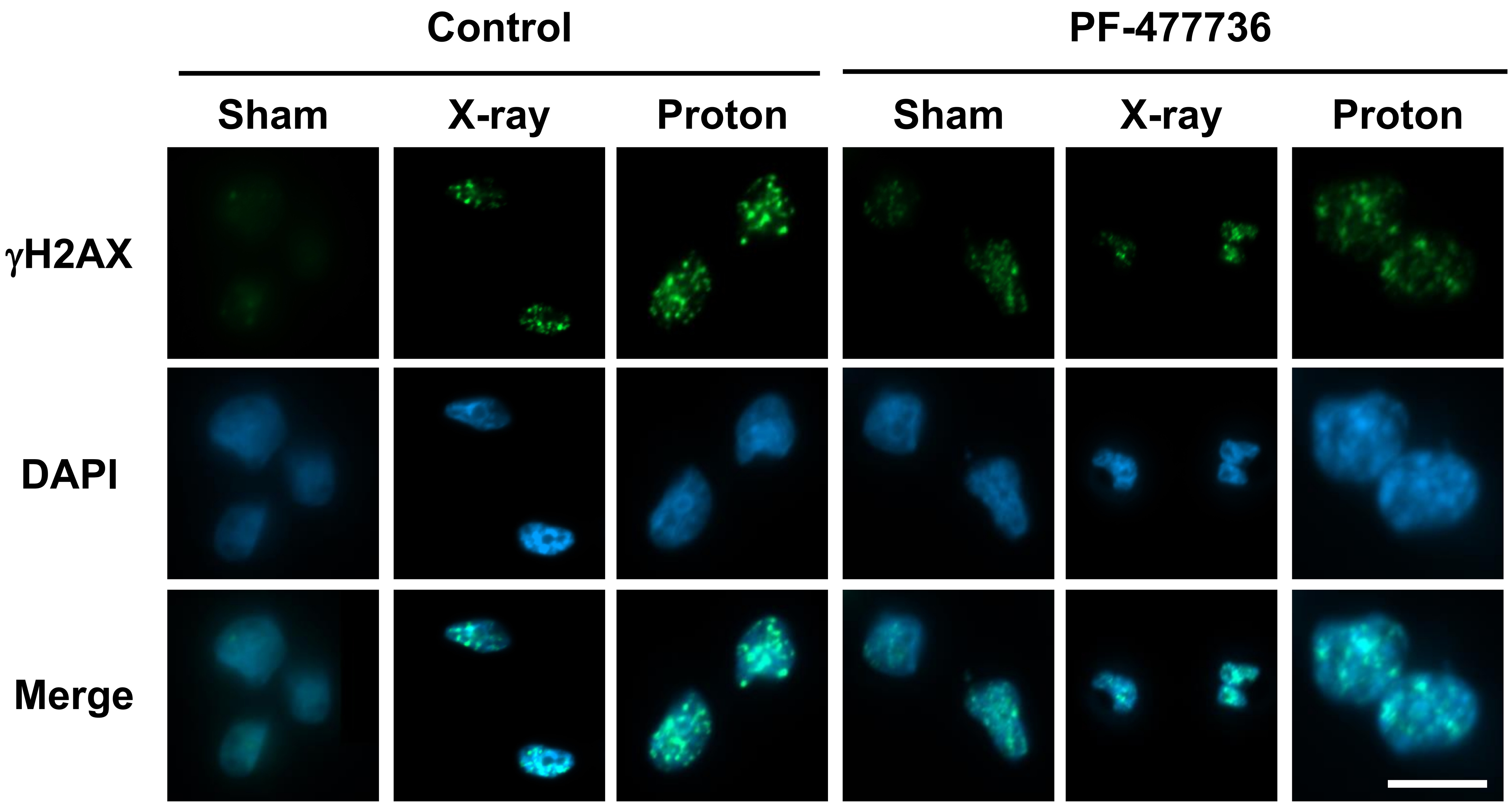

Figure S2. Representative immunofluorescent images showing the effect of PF-477736 on early DNA damage responses after the irradiation with X-rays or protons. MDA-MB-231 cells were pre-treated with 500 nM PF-477736 for 3 h, followed by irradiation with 4 Gy of X-rays or protons. After 30 min, cells were fixed and probed using the γ-H2AX antibody (green) and stained with DAPI (blue). The scale bar indicates 10 μm.

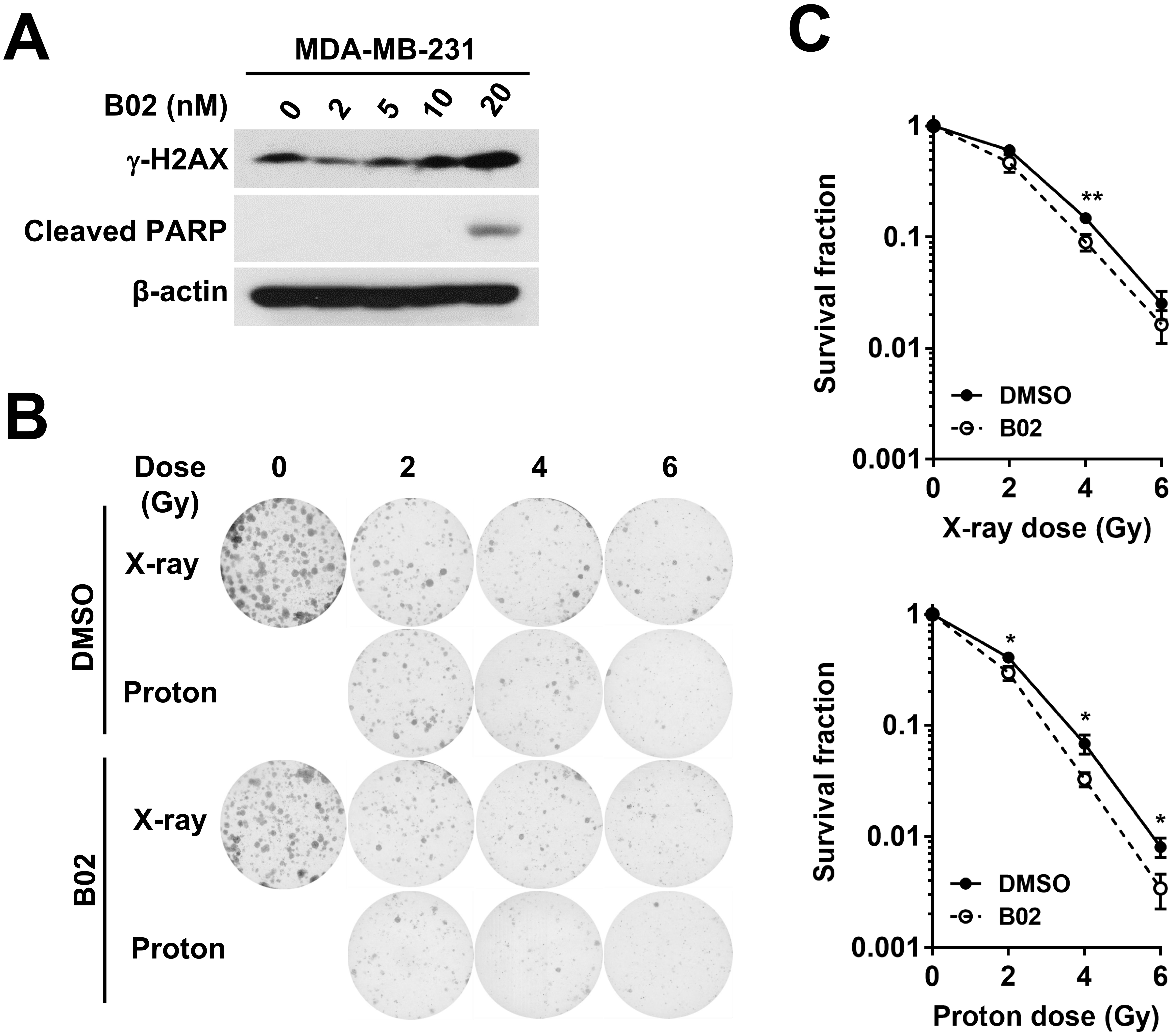

Figure S3. Effects of a Rad51 inhibitor, B02 on MDA-MB-231 cells. (**A**) B02 treatment increased DNA damage and apoptosis in a concentration-dependent manner. (**B**) B02 treatment sensitized MDA-MB-231 cells to radiations. Cells were pre-treated with 2 nM B02 for 3 h, and then were exposed to the indicated doses of X-rays or protons. After 14 days, colonies were stained with crystal violet. (**C**) Survival curves of MDA-MB-231 cells after irradiation showed B02 exerted a stronger radiosensitizing effect with protons than X-rays. *$p<0.05$; **$p<0.01$.
